# The Effect of a Hidden Source on the Estimation of Connectivity Networks from Multivariate Time Series

**DOI:** 10.3390/e23020208

**Published:** 2021-02-08

**Authors:** Christos Koutlis, Dimitris Kugiumtzis

**Affiliations:** 1 Information Technologies Institute, Centre of Research and Technology Hellas, 57001 Thessaloniki, Greece; ckoutlis@iti.gr; 2 Department of Electrical and Computer Engineering, Aristotle University of Thessaloniki, University Campus, 54124 Thessaloniki, Greece

**Keywords:** granger causality, causality networks, multivariate time series, hidden source, finance

## Abstract

Many methods of Granger causality, or broadly termed connectivity, have been developed to assess the causal relationships between the system variables based only on the information extracted from the time series. The power of these methods to capture the true underlying connectivity structure has been assessed using simulated dynamical systems where the ground truth is known. Here, we consider the presence of an unobserved variable that acts as a hidden source for the observed high-dimensional dynamical system and study the effect of the hidden source on the estimation of the connectivity structure. In particular, the focus is on estimating the direct causality effects in high-dimensional time series (not including the hidden source) of relatively short length. We examine the performance of a linear and a nonlinear connectivity measure using dimension reduction and compare them to a linear measure designed for latent variables. For the simulations, four systems are considered, the coupled Hénon maps system, the coupled Mackey–Glass system, the neural mass model and the vector autoregressive (VAR) process, each comprising 25 subsystems (variables for VAR) at close chain coupling structure and another subsystem (variable for VAR) driving all others acting as the hidden source. The results show that the direct causality measures estimate, in general terms, correctly the existing connectivity in the absence of the source when its driving is zero or weak, yet fail to detect the actual relationships when the driving is strong, with the nonlinear measure of dimension reduction performing best. An example from finance including and excluding the USA index in the global market indices highlights the different performance of the connectivity measures in the presence of hidden source.

## 1. Introduction

The construction of networks gives the ability to approach problems in a simple and informative way in many branches of science [1]. In the analysis of multivariate time series in many fields, ranging from neurophysiology to finance, many methods have been developed to measure the interdependence between the components of the observed system and then illustrate it as a graph. Granger causality [2] and variations of this concept have been extensively used to estimate the driving–response relationships between the variables (representing subsystems) of a system, including linear and nonlinear measures on the time, frequency and phase domain, as well as information-based measures (see [3] for a recent comparative study of many connectivity measures). Connectivity measures, referred to as measures of Granger causality, interdependence, coupling and information flow, have been applied in many fields, and mainly in neuroscience and physiology [4,5,6], finance [7,8] and climatology [9,10]. Here, we concentrate on multivariate connectivity measures that estimate the direct causal effect from one observed variable to another accounting for the presence of the other observed variables. Originally, to serve this purpose, the linear vector autoregressive (VAR) model was proposed by Sims [11]. It is also noted that we do not differentiate asymmetric causal effects here, though often observed in applications in finance [12,13]. The computation of a connectivity measure on all pairs of a multivariate time series gives rise to a connectivity network formed by nodes standing for the observed system variables. The network connections are either weighted defined by the causality index estimating the strength of the causality effect or binary when only the existence of the causality effect is of interest.

The multivariate time series may not include observations of all representative variables (nodes) of the system, so that unobserved variables may not be present as nodes in the causality network. These hidden variables may have weak or strong causal effect on some or all the observed variables and therefore introduce redundant information transfer in the system [14]. Some attempts have been made to account for latent effects in causal models, e.g., for biological systems [15] and financial systems [16], or instantaneous effects as these are signatures of hidden source [17,18]. The effect of information deficiency regarding hidden variables and their connections, on the estimation of the causality connections among the observed variables is an open issue [19]. In an attempt to address this issue, Granger causality measures that account for latent variables were developed assuming correlated error terms in the vector autoregressive model, such as the partial Granger causality index (PGCI) [20]. Alternatively, it is suggested to expand the vector autoregressive models to include instantaneous and lag effects [21], and recently we followed a similar approach in the information theory setting [22]. The effect of hidden variables has been studied on exemplary low dimensional linear systems [23,24,25], employing linear causality measures on the time and frequency domain. For complex systems with many variables it is important to investigate the impact of having a hub variable unobserved on the estimation of connectivity networks, and this is the objective of this work.

In this work, we investigate the robustness in estimating the causality structure among the *K* subsystems of a system when the K+1 subsystem is not observed. In particular, we are interested in cases that the unobserved subsystem is a hub driving the *K* other subsystems. Moreover, we consider the setting of high-dimensional systems (larger *K*) and relatively short time series. For this, we conduct a simulation study using four simulated systems, two nonlinear dynamical systems in continuous and discrete time, the coupled Hénon maps system (CHM) and the coupled Mackey–Glass system (CMG), respectively, and two stochastic systems in continuous and discrete time, the neural mass model (NMM) and the vector autoregressive process (VAR), respectively. Each of the four systems comprises K=25 coupled subsystems or variables and different regimes are determined by the free parameters of each system. Several scenarios are studied for zero, weak and strong coupling between the observed variables, as well as zero, weak and strong driving of the hidden variable to the observed variables. For the direct causality estimation and the formation of the causality networks three conceptually different multivariate causality measures are employed, the PGCI designed for latent variables [20], a linear model-based measure termed RCGI [26] and an information-based measure termed PMIME [27], where the latter two make use of dimension reduction. In addition, an example from finance is illustrated where the causality network of K=23 developed markets is measured with and without the USA market, presumably acting as a driving hub.

The paper is organized as follows. In Section 2, the causality measures and the dynamical systems are presented, in Section 3, the results are illustrated, and in Section 4, conclusions are given.

## 2. Material and Methods

**Figure 1:**
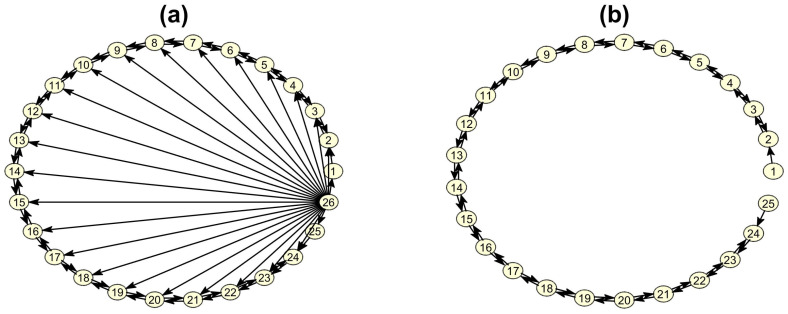
(**a**) The coupling structure of all 25 + 1 subsystems of each dynamical system generating the time series, where the 26th subsystem is the hub source. (**b**) The coupling structure of the 25 observed subsystems, excluding the unobserved source.

In this study, we considered systems of K+1=25+1 subsystems (variables for the VAR system, see below), where one of the subsystems has the role of the source and acts upon the evolution of all other *K* subsystems. Actually, the interest is on studying the system on the basis of K=25 observed subsystems assuming the source is hidden. The particular size K+1 of the system was chosen to balance the high dimension of the observed system and the computational efficiency in the simulations (in the sequel we use the notation *K* practically being 25 in all simulations). For each of the simulated systems, the *K* subsystems are coupled in a simple open ring format, each one driving its left and right neighbor, while the first and *K*th subsystem drive only their sole neighbor, and all directed couplings have the same strength $C_{ij}=C$, i,j=1,…,K. The (K+1)th subsystem acts as a hub source, driving all other subsystems (see Figure 1a) with the same coupling strength $C_{K,j}=S$.

This is the unobserved subsystem in our simulations as we do not include it in the causality analysis. Thus the subnetwork of the *K* observed subsystems (see Figure 1b) is assumed as the true network of *K* subsystems to be matched by the estimated network on the *K* time series of representative variables from each subsystem. This is indeed an assumption, and in general additional connections among the observed subsystems may occur but at smaller strength if one attempts to recast the equations of the system replacing the hidden subsystem in the equations by its expression with respect to the observed subsystems (see [22]).

**Figure 2:**
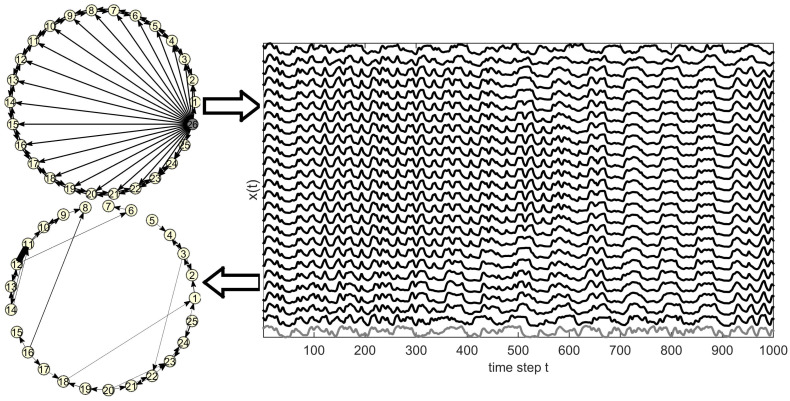
The upper network shows the initial coupling structure (the gray colored node denotes the hidden source) which yields the multivariate time series shown in the graph to the right (the gray colored time series at the bottom is of the hidden source). The lower network shows the estimated causality between the variables of the system when the source is not observed.

The problem of estimating the true coupling between the observed variables when the hub (source) variable is not observed is illustrated in Figure 2.

The original network is formed by the coupling between the *K* observed variables and the (K+1)th source variable driving all *K* variables. The upper arrow points from the original network to the multivariate time series generated from the system designed by the original network (here the CMG system, to be presented below). The lower arrow points from the multivariate time series to the network estimated by a causality measure (here the PMIME to be presented below) on the *K* observed time series (excluding the source time series), which has to be compared to the original network restricted to the *K* observed variables. In order to assess how well the estimated causality network matches the original network restricted to the *K* observed variables, we compute the performance indexes of sensitivity and specificity. Sensitivity is the proportion of the true connections present in the estimated network, given as Sens = TP/(TP + FN), where true positives (TP) are the true causal relationships correctly identified as such and false negatives (FN) are the true but undetected connections. Specificity is the proportion of non-existing connections being also not present in the estimated network, given as Spec = TN/(TN + FP), where true negatives (TN) are the connections correctly not detected and false positives (FP) are the falsely detected connections.

The causality measures and simulated systems used in the study are briefly presented below.

### 2.1. Causality Measures

We included three direct causality measures with different properties. The first causality measure relies on linear vector autoregressive (VAR) models designed to address the presence of latent variables [20], and thus it is particularly relevant for our case with hidden source. The two other measures are designed for high-dimensional time series (large *K*) and make use of dimension reduction, the one using restricted VAR models and the other selecting the causal lagged variables using information criteria. All three measures estimate the direct causal effect of an observed variable *X*, the driving variable, on another observed variable *Y*, the response variable. The effect is direct because it is estimated in the presence of all other K−2 observed variables, denoted collectively *Z*.

The measure partial Granger causality index (PGCI) inspired by the definition of partial correlation is based on VAR models similarly to the measure of *conditional Granger causality index* (CGCI) that applies the Granger idea in multivariate time series [20]. As for CGCI, two VAR models of order *p* are considered, the unrestricted model (U-model) containing all lagged terms for *X*, *Y* and *Z* (up to a given maximum lag or order *p*) and the restricted model (R-model) excluding the lagged terms of *X* from the U-model. Unlike CGCI, two additional error terms are added in the two models, one representing exogenous inputs and another representing latent variables. This modification results in a different expression than the standard log ratio of the fitted error variances of U-model and R-model in CGCI. The derived PGCI is meant to measure the direct causality as CGCI but accounting also for the presence of exogenous and latent variables.

The measure restricted conditional Granger causality index (RCGCI) is an adaptation of CGCI for high-dimensional time series restricting the VAR U-model used in CGCI to the most explanatory terms for the response *Y* (one time step ahead) [26]. The terms of the standard VAR U-model are all the lagged variables up to a maximum lag (order) *p* so that in total there are Kp terms. Applying a selection scheme using augmented VAR models, called modified backward-in-time selection, a small subset of lagged variables are selected in the restricted VAR model to constitute the U-model for RCGCI. For high-dimensional systems with sparse coupling structure, as the open ring structure in Figure 1, the selected subset may have cardinality much smaller than Kp. The restricted model (R-model) is derived by excluding the lagged terms of the driving variable *X*, so that if the restricted VAR U-model contains no lagged terms of *X* then RCGCI = 0. If it contains lagged terms of *X* then RCGCI is computed by the log ratio of the fitted error variances of the U-model and R-model as for the CGCI. Though RCGCI is a linear measure, it was recently found to perform well on high-dimensional nonlinear systems in [3], and it was therefore included in this study.

The measure partial mutual information from mixed embedding (PMIME) applies dimension reduction by selecting the most relevant lagged variables to the response *Y* (one time step ahead, $y_{t+1}$) from the pool of all the Kp lagged variables as for RCGCI, but using information criteria [27]. In particular, in the first step of the selection scheme, the lagged variable *w* with the highest mutual information (MI) to the response, $I(y_{t+1};w)$, is selected and constitute the current subset of lagged variables $w_{t}$. For each subsequent step, the lagged variable *w* with the highest conditional mutual information to the response given the current subset, $I(y_{t+1};w|w_{t})$, is selected and added to $w_{t}$. The significance of the conditional mutual information (mutual information for the first step) is tested at each step and the selection scheme terminates when it is found statistically not significant. Similarly to RCGCI, if the derived subset does not include any lagged terms of the driving variable *X* then PMIME = 0. If it includes the information of the lagged terms of *X*, the response is evaluated by the conditional mutual information of the lagged terms of *X* in $w_{t}$, $w_{t}^{x}$, and the response given all other components in $w_{t}$, normalized by the mutual information of all the lagged terms and the response

(1) $$\mathrm{PMIME}=\frac{I(y_{t+1};w_{t}^{x}|w_{t}^{y},w_{t}^{z})}{I(y_{t+1};w_{t})},$$

where $w_{t}^{y}$ and $w_{t}^{z}$ are the lagged terms of *Y* and *Z* in $w_{t}$, respectively, so that $w_{t}=\left[w_{t}^{x},w_{t}^{y},w_{t}^{z}\right]$. We note that the numerator in (1) is similar to the conditional mutual information defining the transfer entropy (TE) for the bivariate case (X,Y) [28] and the partial transfer entropy (PTE) (X,Y,Z) [29], but these measures use all the lagged variables which in this case would require the estimation of entropy terms (defining the mutual information) of variables of dimension up to Kp+1 (Kp lagged variables and the response). Thus, in our setting of high-dimensional time series (here K=25), the PTE would not give sensible results.

### 2.2. Simulated Systems

The main aim of the simulation study was to assess the estimation of the connectivity structure with the three causality measures when an important source is not observed. For a more comprehensive assessment, different systems and data settings are considered. In particular, four high-dimensional systems are considered and their predefined connectivity structure is the ground truth to which the estimated connectivity structure is compared: the coupled Hénon maps (CHM) [27], the coupled Mackey–Glass (CMG) [27,30], the neural mass model (NMM) [31] and the vector autoregressive model (VAR) as suggested in [32], presented briefly below. All four systems comprise K=25 subsystems (single variables for the last system) coupled in an open ring format plus one more subsystem (considered not to be observed) driving the other *K* subsystems, as shown in Figure 1. The strength is fixed for the coupling of any pair of the observed subsystems and the same holds for the driving of the hidden source to each of the observed subsystems. However, different scenarios are considered for the level of the fixed coupling and driving strengths, and also for the regime of the second and third system by changing a system parameter. For each scenario 10 realizations are made to obtain statistically stable results.

**CHM** The system of chaotic coupled Hénon (CHM) maps is defined as (see also [22])

$$x_{j,t}=1.4-{\left[\frac{\sum_{i=1}^{K+1}C_{ij}x_{i,t-1}}{\sum_{i=1}^{K+1}\Theta \left(C_{ij}\right)}+\left(1-\frac{\sum_{i=1}^{K+1}C_{ij}}{\sum_{i=1}^{K+1}\Theta \left(C_{ij}\right)}\right)x_{j,t-1}\right]}^{2}+0.3x_{j,t-2}$$

where j=1,2,…,K+1 is the variable index, *K* denotes the number of the observed subsystems and $C_{ij}$, i,j=1,2,…,K+1 and i≠j, is the coupling strength (considering $x_{i}$ as the driving variable and $x_{j}$ as the response variable). The connectivity structure of the first *K* observed subsystems is of an open ring type, where the first subsystem drives the second and the *K*th subsystem drives the (K−1)th subsystem and all others drive the one before and the one after, and all driving strengths are equal to *C*. The K+1 subsystem is the unobserved source, not driven by any of the *K* subsystems but driving all *K* subsystems with the same strength *S* (see Figure 1a). We consider three levels of coupling strength among the observed variables, no-coupling, weak coupling and strong coupling assigned to C=0,0.15,0.3, respectively. Accordingly, we consider the strengths of the source driving CS=0,0.1,0.3. The time series length is set to L=400.

**CMG** The coupled Mackey–Glass system is a system of coupled identical delayed differential equations defined as

(2) $${\dot{x}}_{j}\left(t\right)=-0.1x_{j}\left(t\right)+\sum_{i=1}^{K+1}\frac{C_{ij}x_{i}\left(t-\Delta \right)}{1+x_{i}{\left(t-\Delta \right)}^{10}}$$

where the notation and coupling connectivity structure is as for CHM, but here $C_{jj}=0.2$, j=1,…,K+1. The lag parameter Δ determines the complexity of the inherent dynamics of each subsystem. In the absence of coupling, each subsystem gives rise to a chaotic dynamical system (for Δ≥17) of increasing complexity with Δ, and we consider a regime of relatively low complexity Δ=20 and high complexity Δ=100. The three levels of zero, weak and strong coupling strength are assigned here to C=0,0.05,0.1, respectively, and for the same levels the source driving is S=0,0.04,0.1, respectively. The time series length is set to L=1000.

**NMM** The neural mass model is a system of coupled stochastic differential equations that produces time series similar to electroencephalogram simulating a variety of brain activity e.g., normal and epileptic and it is defined as

(3) $$\begin{array}{cc} {\dot{y}}_{0}^{j}\left(t\right)= & y_{3}^{j}\left(t\right)\\ {\dot{y}}_{3}^{j}\left(t\right)= & AaS\left[y_{1}^{j}\left(t\right)-y_{2}^{j}\left(t\right)\right]-2ay_{3}^{j}\left(t\right)-a^{2}y_{0}^{j}\left(t\right)\\ {\dot{y}}_{1}^{j}\left(t\right)= & y_{4}^{j}\left(t\right)\\ {\dot{y}}_{4}^{j}\left(t\right)= & Aa\left\{p^{j}\left(t\right)+C_{2}S\left(C_{1}y_{0}^{j}\right)+\sum_{\begin{array}{c} i=1\\ i\neq j \end{array}}^{K+1}C_{ij}y_{6}^{i}\left(t\right)\right\}\\ \; & -2ay_{4}^{j}\left(t\right)-a^{2}y_{1}^{j}\left(t\right)\\ {\dot{y}}_{2}^{j}\left(t\right)= & y_{5}^{j}\left(t\right)\\ {\dot{y}}_{5}^{j}\left(t\right)= & Bb\left\{C_{4}S\left[C_{3}y_{0}^{j}\left(t\right)\right]\right\}-2by_{5}^{j}\left(t\right)-b^{2}y_{2}^{j}\left(t\right)\\ {\dot{y}}_{6}^{j}\left(t\right)= & y_{7}^{j}\left(t\right)\\ {\dot{y}}_{7}^{j}\left(t\right)= & Aa_{d}S\left(y_{1}^{j}\left(t\right)-y_{2}^{j}\left(t\right)\right)-2a_{d}y_{7}^{j}\left(t\right)-a_{d}^{2}y_{6}^{j}\left(t\right) \end{array}$$

where *j* denotes the neuron population represented by eight interacting variables and the population interacts with other populations through variable $y_{4}^{j}$ with coupling strength $C_{ij}$. The notation and coupling connectivity structure is as for CHM. The term $p^{j}\left(t\right)$ is the stochastic input and represents a random influence from neighboring or distant populations, *A* is an excitation parameter and *B*, *a*, *b*, $a_{d}$, C1−C4 other parameters (see [31] for more details). The function *S* is the sigmoid function $S\left(v\right)=2e_{0}/\left(1+e^{r(v_{0}-v)}\right)$, where *r* is the steepness of the sigmoid and $e_{0}$, $v_{0}$ are other parameters explained in [31]. From each population we keep the same variable, e.g., the 1st out of 8, and so we obtain multivariate time series with K+1 variables. The value of the excitation parameter *A*, affects the form of the output signals combined with the coupling strength level, ranging from similar to normal brain activity with no spikes to almost periodic with many spikes similar to epileptic brain activity. Two scenarios are considered for this system regarding A=3.6,3.9, and the zero, weak and strong coupling are given by C=0,80,200 for the coupling among the *K* observed variables and S=0,70,200 for the source driving. The time series length is set to L=1000.

**VAR** The vector autoregressive process (VAR) order p=1 is defined on K=25+1 variables. The coupling connectivity structure is as for CHM setting to zero the coefficients of the model terms that do not correspond to causal effects. Initially, the coefficients of the existing causal effects are set to 0.9 and they are reduced iteratively until the stationarity condition is fulfilled at a level *c* (as suggested by the authors in [32]). For this system we consider two cases of zero and weak coupling given by C=0,c for the coupling among the *K* observed variables and S=0,c for the source driving. The time series length is set to L=400.

### 2.3. Real Data

**Figure 3:**
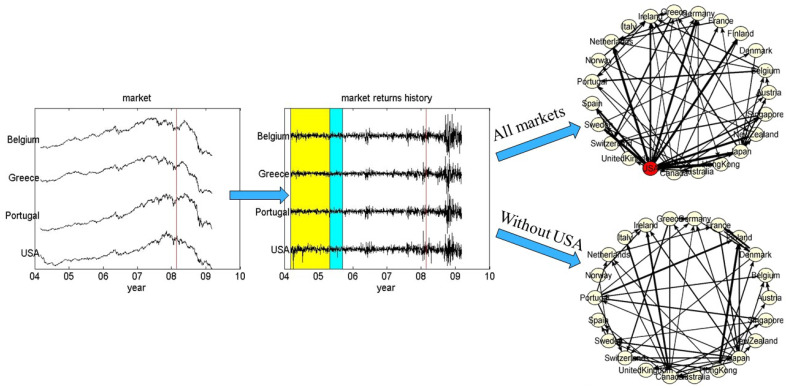
The two panels at the left show the prices and the returns, respectively, for four of the 23 markets. In the second panel, the time window and sliding step are shown with the shaded areas (yellow and blue online, respectively). The upper network is estimated on the basis of all 23 markets, while the lower network is on all but USA markets.

For the real data example, we consider financial time series from Morgan Stanley Capital International (MSCI) of daily indices for 23 developed national markets from March 2004 to March 2009. The data are transformed to returns (see Figure 3).

The whole period is split to 11 overlapping windows of length 300 and step 100 time points. Causal interactions are estimated for all windows in order to compute and observe the changes of the node strength of each developed market in time. We consider the USA index as the hidden source variable (driving hub) and we measure the causality with and without it as if it was not observed.

Finally, we discuss the free parameter of model order and maximum lag *p* in PGCI, RCGCI and PMIME. We do not use model order selection criteria, such as the Bayesian information criterion, but use instead a sufficiently large order *p*. In the simulations, the selected *p* is larger than the true order of the system, call it $p_{0}$. Concretely, for the VAR generating system with $p_{0}=1$ we use p=2 for the computation of PGCI, RCGCI and PMIME. Likewise, the coupled Hénon maps (CHM) are defined for maximum lag 2, which suggests $p_{0}=2$, and the selected order and maximum lag in PGCI, RCGCI and PMIME is p=5. For the continuous-time systems, the coupled Mackey–Glass (CMG) and the neural-mass model (NMM), there is no original order $p_{0}$ determined by the system equations, as they are defined in continuous time. To be on the safe side, we chose a sufficiently large p=60. In this way, we assured that any causal lag-terms were included in the computation of the causality measures. Simulations have shown that RCGCI and PMIME are rather insensitive to *p* as long as this is sufficiently large to include all possible lag causal terms ($p\geq p_{0}$) [26,27]. This is so, because both measures are derived by an iterative algorithm that selects the causal lag terms among all the candidate lag terms up to the given order. The PCGCI may be more sensitive to the order *p*, but again we expect that as long as the order is sufficiently large, the terms of higher lag will be found statistically not significant when running the significance test. The preselection of order as opposed to using a selection criterion allows for using the same parameter setting in the computation of the causality measures across different realizations (simulations and windowing for real data).

## 3. Results

**Figure 4:**
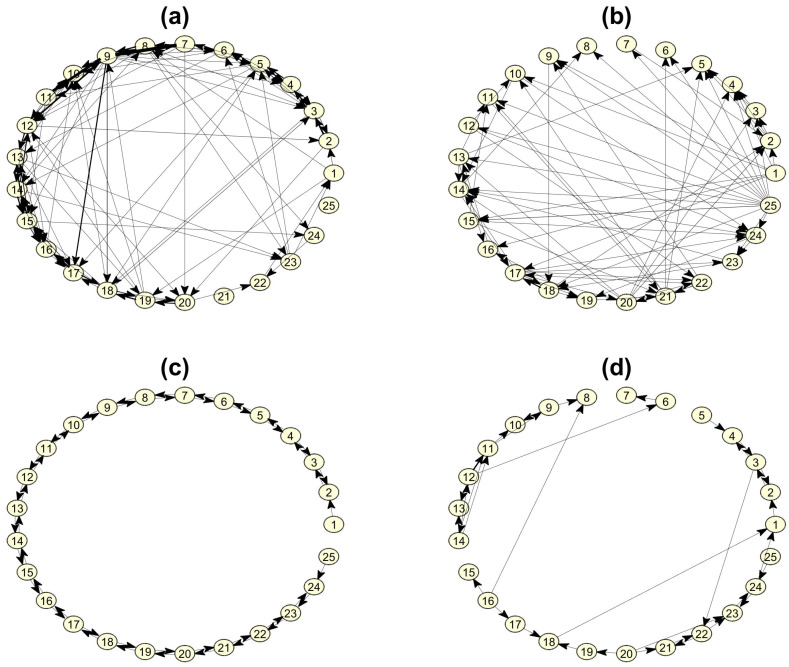
Exemplary estimated connectivity networks for weak and strong source driving: (**a**) restricted conditional Granger causality index (RCGCI) networks for coupled Hénon maps system (CHM), C=0.3 and S=0.1. (**b**) RCGCI network for CHM, C=0.3 and S=0.3, (**c**) PMIME network for coupled Mackey–Glass system (CMG), C=0.1 and S=0.04. (**d**) PMIME network for CMG, C=0.1 and S=0.1.

Examples of the estimated networks for the simulated systems are shown in Figure 4.

First, we show that the RCGCI on the system CHM detects the open ring form of the connectivity structure and estimates correctly most of the true causal effects but adds also many spurious causal effects when the hidden source driving is weak (comparing the estimated network in Figure 4a to the true network without the source and its connections in Figure 1b. The spurious causal effects are expected since RCGCI is a linear measure. However, when the source driving is strong it fails to detect the true connectivity structure and the estimation of coupling is merely random (comparing Figure 4b to Figure 1b. In another example, PMIME on the system CMG estimates exactly the true connectivity structure for weak source driving (comparing Figure 4c to Figure 1b but misses a good proportion of true causal effects (and adds some spurious causal effects) when the source driving gets strong comparing Figure 4d to Figure 1b. The two examples give some evidence for good performance of the causality measures when the hidden source driving is weak which worsens when it gets strong. Quantitative summary results are given below from 10 realizations for each setting.

**Figure 5:**
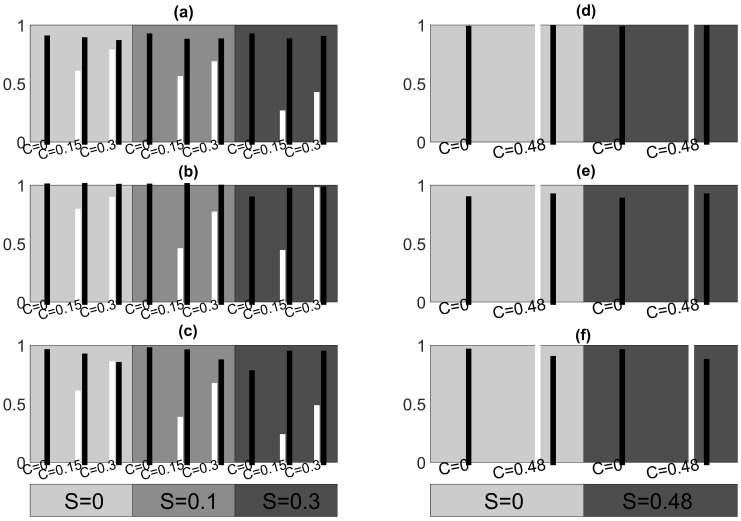
Sensitivity (white), specificity (black) for different *C* and *S* (the gray level of the shaded areas increase with *S*). For CHM: (**a**) RCGCI, (**b**) PMIME, (**c**) partial Granger causality index (PGCI). For vector autoregressive (VAR): (**d**) RCGCI, (**e**) PMIME, (**f**) PGCI.

**Figure 6:**
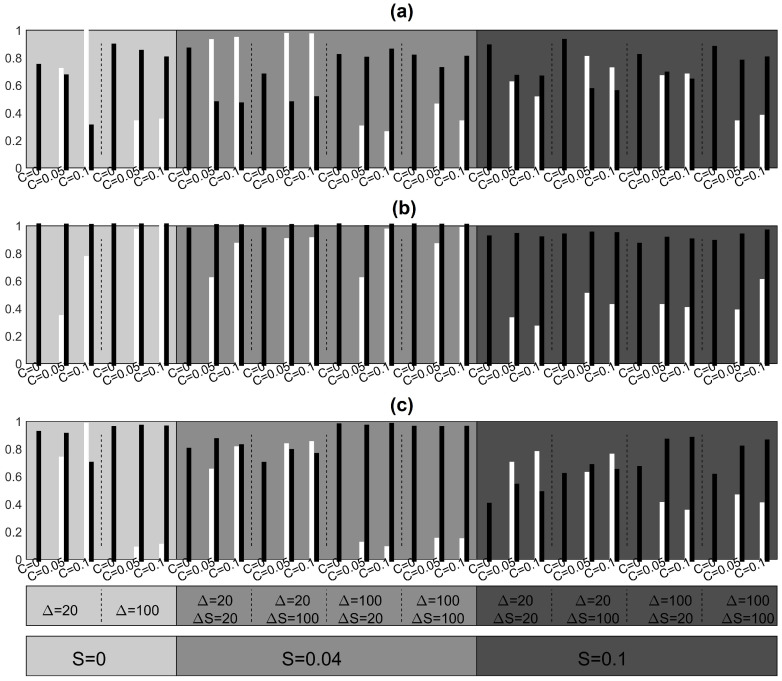
Sensitivity (white), specificity (black), for CMG in two complexity levels (Δ=20,100) and different *C* and *S* (the gray level of the shaded areas increase with *S*): (**a**) RCGCI, (**b**) PMIME, (**c**) PGCI.

**Figure 7:**
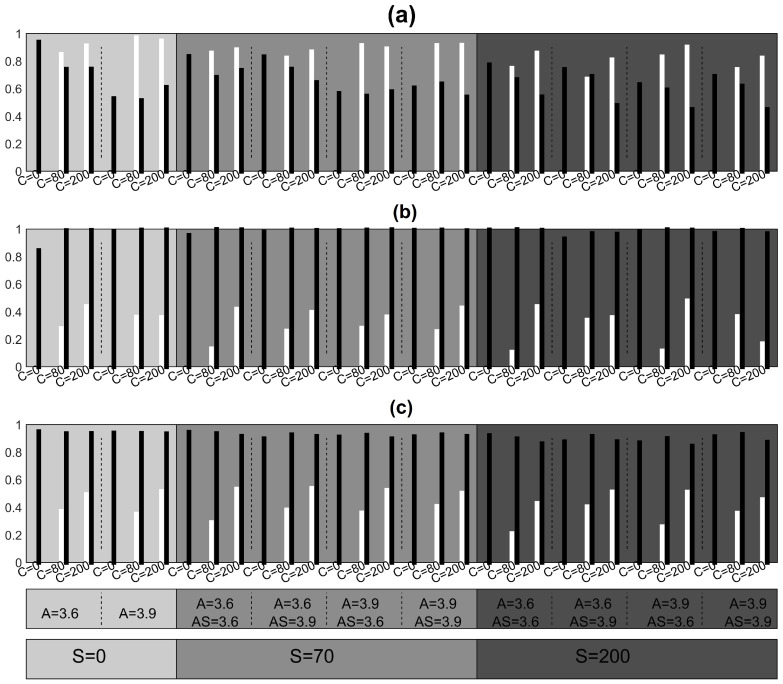
Sensitivity (white), specificity (black), for neural mass model (NMM) in two complexity levels (A=3.6,3.9) and different *C* and *S* (the gray level of the shaded areas increase with *S*): (**a**) RCGCI, (**b**)PMIME, (**c**) PGCI.

We consider as ground truth the initial coupling structure without the source and its connections in order to evaluate the performance of the measures in terms of sensitivity and specificity. Summary results on sensitivity and specificity for all settings of the simulation study are shown in Figure 5 for systems CHM and VAR, Figure 6 for system CMG and Figure 7 for system NMM.

For the CHM system in Figure 5, PMIME exhibits the highest sensitivity and specificity for all combinations of *C* and *S*. It always attains very high specificity while the lowest sensitivity is for weak coupling among the observed variables (C=0.15) in the presence of source driving (S=0.1,0.3). In particular, for the highest coupling strengths C=0.3 and S=0.3, the PMIME estimates perfectly the true connectivity network, better than for weak S=0.1, missing around 20% of true connections (smaller sensitivity, see Figure 5b). The linear measures perform also well in this system but attain smaller specificity and sensitivity than PMIME with PGCI having smaller specificity than RCGCI. For the VAR system in Figure 5, the RCGCI gives perfect reconstruction of the coupling structure in all realizations as expected. In addition, the PMIME and PGCI show the same great performance regarding sensitivity, but they detect some spurious extra causality effects as well. For the CMG system in Figure 6, in most of the cases PMIME shows generally better performance attaining always the highest specificity (most of the cases close to 1), but also in some cases the other two causality measures achieve the same or even better sensitivity.

For the NMM system in Figure 7, the RCGCI has the best performance with high sensitivity level but lower specificity as compared to PMIME and PGCI. The PMIME and PGCI show similar performance with low sensitivity and high specificity.

Focusing on specificity with respect to the CHM system, we observe that RCGCI exhibits the same performance regardless of the strength *C* and *S*, while for PGCI and also in some extent for PMIME it is significantly reduced when there are no connections among the observed variables (C=0). For the VAR and NMM systems, when C=0 the specificity is preserved in the same level for all three causality measures and for different hidden source driving levels. On the other hand, for the CMG system, the specificity is comparatively low for the RCGCI and PCGI and decreases with strong source driving. The latter also holds for PMIME.

Sensitivity is reduced when the source has a strong impact on the observed variables and this is because the introduced correlations among the observed variables mask partially the information flow. In addition, the sensitivity increases with the coupling strength *C* between the variables in most cases, while the specificity is preserved at the same level for all causality measures. Few false connections are detected by the PMIME when a strong hidden source is present while in its absence it achieves perfect performance for systems CHM, VAR and CMG. In addition, the PMIME estimates almost perfectly the true network in CMG case when a hidden source is absent or weak but when it is strong many true relationships are not detected. For the system CHM, the PMIME has decreased sensitivity when the coupling among the observed variables is weak but achieves almost perfect performance when it is strong. The RCGCI, as a linear measure, shows worse performance both for sensitivity and specificity compared to that of the PMIME in all scenarios of systems CHM and CMG. For almost all settings of the system CHM, the network estimation by the PMIME is in overall worse than for CMG. In general terms, the measures show a good performance except for the NMM where very few causality effects are detected but at least most of them are true as we can infer from Figure 7.

**Figure 8:**
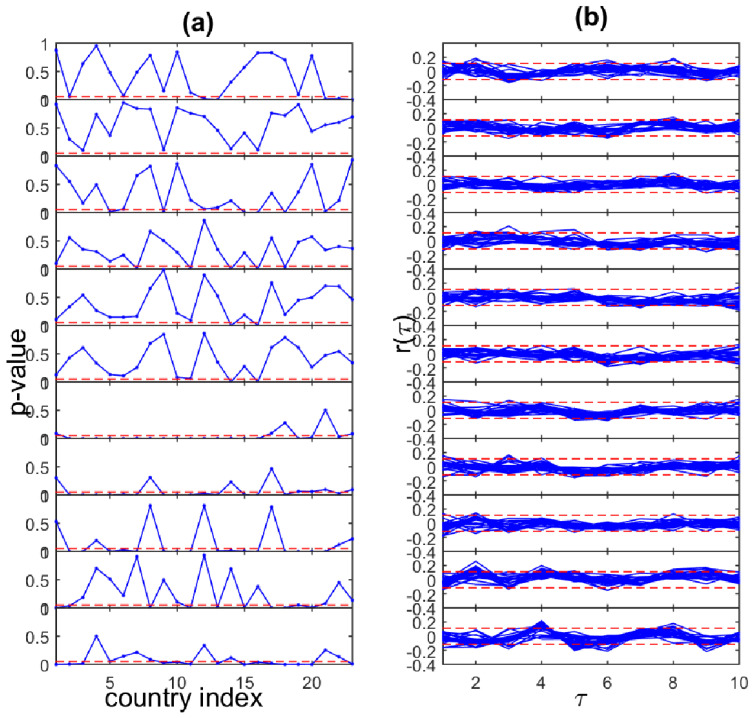
(**a**) The *p*-value of the Engle test for heteroscedasticity on the return time series vs. the country index for the 11 sliding windows (panels from top to bottom). The significance level of α=0.05 is shown with stippled horizontal line at each panel. (**b**) The autocorrelation vs. lag for the return time series of the 23 countries for the 11 sliding windows (panels from top to bottom). The significance levels for the autocorrelation $\pm 1.96/\sqrt{300}$ are shown with stippled horizontal lines at each panel.

In the financial application, we observe that the return time series for the 23 developed markets are overall stationary in variance in the 11 sliding windows of length n=300. This is confirmed by the Engle test for heteroscedasticity [33], where statistically significant heteroscedasticity effects are found for only some windows for the majority of the countries (windows 7, 8 and 9, as shown in Figure 8a).

We point out this possible inadequacy, i.e., violation of the assumption of stationarity due to heteroscedasticity in a small subset of the tested return time series, but proceed with the same setting of analysis for all the time series. With regard to the model order and maximum lag *p*, we observe that the return time series have only very weak autocorrelation, if any, at small lags, as shown in Figure 8b. Therefore, we set a sufficiently large order p=5 in the computation of RCGCI and PMIME.

**Figure 9:**
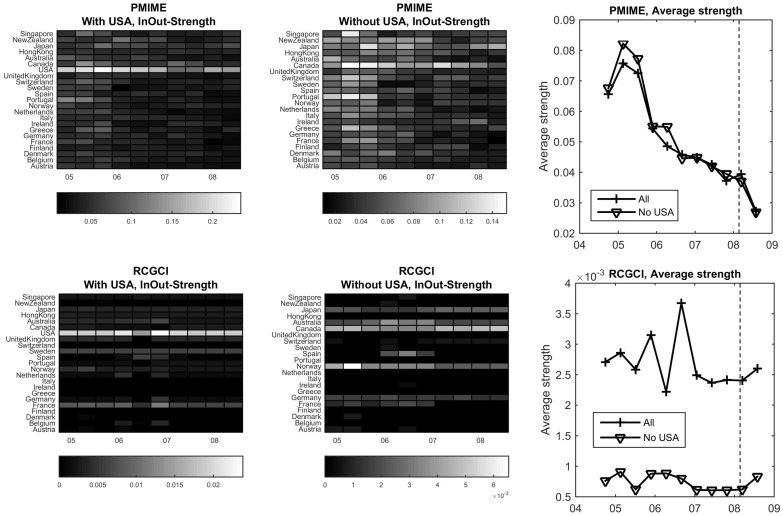
First row: PMIME, second row: RCGCI. At each row, the first two panels show the strength of each market as a function of time with (first) and without (second) USA, and the third plot shows the average strength of the causality networks vs. time with and without USA, where the vertical line denotes the financial crisis in the beginning of 2008.

Both the PMIME and RCGCI detect USA as a hub driving all other markets. This can be seen by the node strength as a function of time, the sliding time windows of 300 days. As shown in Figure 9 (first panel of each row), the coupling strength of USA is much larger than for any other market (the same can be seen restricting to the out-strength, summing the directed coupling from each node to all other nodes).

In the absence of USA, having the role here of the hidden source, Canada tends to show increased strength across the time windows as it has similar evaluation to USA, as shown in Figure 9 (second panel of each row). This is more clearly shown using PMIME as for RCGCI other markets (Norway, Japan and partially Australia, Germany and France) have also large coupling strength across the time windows. It is noted that for RCGCI, when including USA also other markets (France, Sweden) show distinct coupling strength across the time windows. The PMIME detects a decreasing strength trend towards the financial crisis of 2008 while RCGCI does not recognize such a trend. This can be better seen from the average strength over all nodes (markets), as shown in Figure 9 (third panel in each row). Another remarkable note is that for the PMIME in the absence of the hub variable of USA index, all countries show larger strength than in its presence. As a result of this, the average strength is at the same level with and without USA, whereas for the RCGCI the average strength is dramatically decreased in the absence of USA.

## 4. Discussion

In this work, we investigated the ability of causality measures to detect the true coupling structure among the observed variables of coupled subsystems driven also by an unobserved subsystem acting as a hidden source. The interest was particularly on high-dimensional systems, typically met in the study of complex systems in finance, climate and neuroscience, and the hidden source may disguise the true coupling structure of the observed subsystems. We assessed the preservation of the estimated coupling structure when the source was removed from the observations, the multivariate time series. For this, we conducted a simulation study using two high-dimensional nonlinear dynamical systems, the coupled Hénon maps system and the coupled Mackey–Glass system, as well as a stochastic high-dimensional nonlinear system, the neural mass model and a high-dimensional linear stochastic process, the vector autoregressive process. Different settings of the coupling structure of the systems were considered for different levels of the strength of the coupling among the observed variables (given by the parameter *C*) and the hidden source driving (given by *S*). Moreover, three connectivity measures were used, two linear and one nonlinear, all designed to estimate direct causality in the presence of other observed variables besides the driving-response variable pair. The one linear measure, termed PGCI, is designed to account for the presence of exogenous and latent variables, so it is well-suited for our scope. The other two measures, termed RCGCI and PMIME, apply a dimension reduction scheme in the estimation of direct causality and they are thus suitable for high-dimensional systems serving also the scope of this study. The causality networks from each measure are compared to the initial causal network confined on the observed variables (excluding the unobserved source variable) in terms of sensitivity and specificity.

In the simulation study, we establish that when the driving of the hidden source is weak the correct estimation of the coupling structure of the observed variables is still possible. However, when the driving is strong, the measures fail to detect existing relationships and find as significant others that are non-existent. In addition, it is observed that the nonlinear measure PMIME performs best, as expected, on the nonlinear dynamical systems under study. However, RCGCI being a linear measure has great performance especially in the case of the VAR system and also in the case of CHM and NMM systems. Overall, the linear measures are most affected by the presence of the hidden source (S>0) tending to detect more false couplings (lower specificity), while the nonlinear measure PMIME is rather stable in terms of specificity.

The PCGCI designed for latent variables and subsequently hidden source does not perform any better than the RCGCI which addresses the high-dimensionality of the time series, meaning here many terms and coefficients to estimate in the VAR models used to define the causality index. Since the PCGCI is defined in terms of the VAR models, the restriction of VAR in the RCGCI could be utilized to improve the applicability of the PCGCI in high-dimensional time series, which is planned for future work. Similarly, the restricted VAR employed in the RCGCI could also be expanded to include instantaneous effects to account for the hidden source. We have followed a similar approach recently to extent the measure PMIME and applied this to electroencephalograms [22].

The example in finance indicates the importance of including in the calculations a latent confounder that affects strongly the whole system. The analysis of country financial markets including and excluding the USA index, acting supposedly as a hidden source, concluded that the USA index has a very significant contribution in the system of worldwide stock markets, which is confirmed by both the linear and the nonlinear connectivity measures. In addition, its absence in the causality analysis gives rise to the contribution of all other indices especially the index of Canada market. Finally, a trend is observed in the average strength of the PMIME towards the financial crisis of 2008, with or without the USA index.

It should be noted that in the assessment of the connectivity estimation when a hub variable is unobserved, we assume as the true network the subnetwork derived by excluding the hidden source node and its connections. Certainly, the direct connections between the observed variables are not affected by the absence of the source variable. Therefore, these connections can indeed be taken as true. However, additional connections may occur when one assumes the new system of the observed variables, excluding from the system the hidden variable. The substitution in the system equations of the hidden variable is likely to result in new connections between the observed variables. However, the resulting connections are expected to have smaller strength because a direct driving from the source is substituted by a driving through intervention of an observed variable. Therefore, we consider that the assumption that the subnetwork confined only on the basis of the observed variable is approximately true and the results on sensitivity and specificity are not substantially affected by some additional but weaker connections.

References yes
